# Neighborhood disorder, exposure to violence, and perceived discrimination in relation to symptoms in midlife women

**DOI:** 10.1186/s40695-018-0043-0

**Published:** 2018-10-19

**Authors:** Linda M Gerber, Lynnette Leidy Sievert

**Affiliations:** 1 Department of Healthcare Policy & Research, Division of Biostatistics and Epidemiology, Weill Cornell Medical College, 402 E. 67th St., LA-231, New York, NY 10065 USA; 2 Department of Medicine, Division of Nephrology and Hypertension, Weill Cornell Medical College, New York City, NY USA; 3 Department of Anthropology, UMass Amherst, Amherst, MA USA

**Keywords:** Menopause, Hot flashes, Aches, Stress, Neighborhood disorder, Violence, Discrimination

## Abstract

**Background:**

Some symptoms at midlife are associated with stress, such as hot flashes, trouble sleeping, headaches, or depressed mood. Hot flashes have been studied in relation to laboratory stressors, physiological biomarkers, and self-reported stress, but less is known about hot flashes in relation to the larger context of women’s lives. This study examined the risk of symptoms in relation to neighborhood disorder, exposure to neighborhood violence, social cohesion and perceived discrimination. We hypothesized that women exposed to more negative neighborhood characteristics and discrimination would be more likely to report hot flashes and other midlife symptoms.

**Methods:**

Participants were black and white women, aged 40 to 60, drawn from a cross-sectional investigation of race/ethnicity, socioeconomic status, and blood pressure in New York City (*n* = 139). Demographic information, medical history, menopausal status, and symptoms were measured by questionnaire. Likert scales were used to measure neighborhood characteristics, specifically, the Neighborhood Disorder Scale, the Exposure to Violence Scale, the Perceived Violence Subscale, the Neighborhood Social Cohesion and Trust Scale, and the Everyday Discrimination Scale. Ten symptoms were included in analyses: lack of energy, feeling blue/depressed, backaches, headaches, aches/stiffness in joints, shortness of breath, hot flashes, trouble sleeping, nervous tension, and pins/needles in hands/feet. Each scale with each symptom outcome was examined using logistic regression analyses adjusting for significant covariates.

**Results:**

Black women reported higher scores on all negative neighborhood characteristics and discrimination, and a lower score on the positive Neighborhood Social Cohesion and Trust. Neighborhood Disorder was associated with feeling blue/depressed, aches/stiffness in joints, and hot flashes, and Perceived Violence was associated with aches/stiffness in joints, after controlling for model-specific covariates. There was a lower risk of backaches with increasing Neighborhood Social Cohesion and Trust score. The Everyday Discrimination Scale was associated with lack of energy. Lack of energy, feeling blue/depressed, aches/stiffness in joints, and hot flashes appeared to be most vulnerable to negative neighborhood context and discrimination.

**Conclusions:**

This study adds to the literature linking neighborhood environments to health outcomes. The associations between negative neighborhood contexts and discrimination with diverse symptoms, and the association between social cohesion and back pain, point to the need to expand analyses of stress to multiple physiological systems.

## Background

Multiple symptoms have been associated with the menopausal transition. Some, such as hot flashes, are clearly associated with hormonal changes [1–4]. Other symptoms, such as joint pain and headaches, may be associated with hormonal changes, but the evidence is less straight forward [5, 6]. Social, rather than hormonal, changes may be responsible for depressed mood or trouble sleeping in some women [7, 8]. Although not well established, certain studies suggest that stress may be associated with hot flashes [3, 9, 10], trouble sleeping, headaches, and depressed mood [11–13].

“Stress” can have multiple meanings, and has been measured in multiple ways. In relation to hot flashes, stress has been measured both inside the laboratory [14–16] and outside of the laboratory in relation to perceived stress scores [3, 9, 10], cortisol levels [17–21], measures of blood pressure [22–25], and C-reactive protein [10, 26]. Missing from these analyses is a consideration of the larger context of women’s lives, specifically at the level of problems in the neighborhood and the social challenge of perceived discrimination.

A broad range of research links neighborhood social and economic environments to the health of residents [27–29]. Neighborhoods with high levels of poverty, violence, and disorder have been associated with detrimental effects on individuals residing in these areas [27, 30, 31]. Stress is related to the chronic difficulties encountered within neighborhoods, and this neighborhood stress has been reported to increase vulnerability to immune disorders and cardiovascular disease [32, 33]. Exposure to events known to elicit stressful emotions such as fear, anger, or depression have been assessed by two subscales (Neighborhood Disorder and Exposure to Violence) of the City Stress Inventory [34]. Studies among caregivers of children with asthma have shown an increase in asthma morbidity and depression in association with increasing levels of perceived violence [35, 36].

Neighborhoods with low levels of social cohesion have been associated with increased rates of depression in the Multi-Ethnic Study of Atherosclerosis (MESA) Study [37], coronary calcification in the CARDIA study [38], and to increased risk of acute myocardial infarction mortality in Scania, Sweden [39]. Additionally, the Jackson Heart Study found that, in disadvantaged neighborhoods, low social cohesion was associated with higher levels of cumulative biological risk among African American men [27].

Racial disparities in health have been posited to be linked to exposures of discrimination [40]. Self-reported unfair treatment or perceived discrimination has been reported to contribute to broad-based morbidity [41, 42]. Brondolo et al. [43] have reported that racial discrimination may also influence cardiovascular disease risk. It has been suggested that among African Americans, the experience of everyday unfair treatment leads to a cumulative biological “wear and tear” (or allostatic load [44]) as measured across 22 biomarkers, representing seven system levels, of biological disintegration [45]. The results of that study, conducted among midlife African Americans, adds to the literature linking the stress of discrimination to effects on multiple downstream physiological systems [45]. There is also evidence from the Study of Women’s Health Across the Nation (SWAN) linking higher levels of discrimination to higher levels of allostatic load [46]. In addition, in SWAN, the Everyday Discrimination Scale was administered at baseline and at each of the 13 follow-up periods. Chronic everyday discrimination was associated with more bodily pain, in fully adjusted models, among African-American, Chinese, and non-Hispanic white women [47]. Higher allostatic load levels, in addition to contributing to increased risk for many health outcomes [48], may also contribute to greater reporting of midlife symptoms among both black and white women during this period of increased vulnerability.

The purpose of the study presented here was to examine the risk of symptoms at midlife in relation to neighborhood disorder, exposure to neighborhood violence, and perceived discrimination among black and white women living in New York City. We focused on hot flashes and night sweats because of previous studies that suggest a relationship between stress and vasomotor symptoms [3, 9, 10]. An additional reason for this focus was the suggestion that hot flashes and night sweats may be markers of cardiovascular disease risk [49, 50]. In addition, we examined other possible symptoms at midlife that could be associated with increasingly negative neighborhood characteristics and levels of discrimination. We hypothesized that women who report higher levels of neighborhood disorder, violence, and increasing experience of personal discrimination would be more likely to report hot flashes and other symptoms at midlife, even after controlling for age, ethnicity, BMI, and menopausal status. To our knowledge, this is the first study of symptoms at midlife among black and white women in relation to neighborhood context, beyond discrimination.

## Methods

The Neighborhood Study of Blood Pressure and Sleep, conducted from September 1999 through July 2003, was a cross-sectional investigation of race/ethnicity, socioeconomic status, and diurnal blood pressure (BP) patterns [18, 51]. Data for this study were drawn from this parent study. Because this study examined both neighborhood characteristics and symptom experience at midlife, these data offer a unique opportunity to test our hypothesis that hot flashes are more frequently reported among those residing in a stressful environment.

Participants were recruited through fliers and word of mouth from Weill Cornell Medical College, Mount Sinai School of Medicine, Harlem Hospital, and North General Hospital using a common protocol and consent form approved by the institutional review committee at each of the four institutions. At recruitment, women were 18 to 65 years old, white or black, had no previous cardiovascular disease, and no major medical problems other than hypertension (*n* = 211). Those who were eligible and chose to participate completed informed consent before initiating study procedures. The analyses here focus on women aged 40 to 60 (*n* = 139) at the time of interview in order to better assess symptoms at midlife; thus, this is a subset of a larger study.

### Data collected

Participants completed a self-administered demographic and medical history questionnaire that included questions about education, smoking habits, and menstruation. Age and race/ethnicity were self-reported. Questions about menopausal status queried the last menstrual cycle, whether menstruation had occurred in the previous 12 months, menstrual regularity, and whether cycles had changed in length. Post-menopausal status was defined as having had at least 12 months of amenorrhea. Peri-menopausal status was defined by missed menstrual periods and significant changes in menstrual cycle regularity and length. Pre-menopausal status was defined as having regular menstruation. These categories were used in lieu of the STRAW+ 10 stages [52] because of the cross-sectional nature of the study and the small number of women in the peri-menopausal group. Because of the small number of women in the peri-menopause category, women were grouped into two groups: pre- vs. peri/postmenopause for analyses. Height and weight were measured twice by a technician. The average of the two measurements was used, and body mass index (BMI) was calculated as weight divided by the square of height (kg/m^2^).

The following Likert scales were used to measure neighborhood characteristics: (1) The Neighborhood Disorder (ND) Scale [34] assessed perceptions of neighborhood disorder with 11 items that served as a subset of City Stress Inventory, scaled as 0–33 (e.g., I heard neighbors complaining about crime in our neighborhood; People in the neighborhood complained about being harassed by police). (2) The Exposure to Violence (EV) Scale, [34], is a 7 items subset of the City Stress Inventory, scaled as 0–21 (e.g., A family member was attacked or beaten; A friend was robbed or mugged). (3) The Perceived Violence (PV) Subscale is from the Project on Human Development in Chicago Neighborhoods: Community Survey, 1994–1995 [53], scaled 5–20, (e.g., During the past 6 months, how often was there a fight in this neighborhood in which a weapon was used; How often were their sexual assaults/rape). (4) The Neighborhood Social Cohesion and Trust (NSCT) Scale is a subscale of the Collective Efficacy instrument used to assess social cohesion among neighbors with 5 items, scaled 0–15 (e.g., This is a close-knit neighborhood; People around here are willing to help their neighbors) [54]. (5) The Everyday Discrimination Scale (EDS), scaled 0–45 [55] is a scale of 9 items that assesses chronic and routine experiences of unfair treatment (e.g., You are treated with less courtesy than other people; people act as if they are afraid of you; you are called names or insulted.)

Symptoms associated with menopause were queried with a frequently used questionnaire that embeds menopausal symptoms into a list of everyday complaints [56, 57]. Each participant was asked whether or not she had been bothered by each of 23 symptoms during the past 2 weeks, e.g., hot flashes, trouble sleeping, or feeling blue or depressed. Answers were assessed as yes/no.

We selected symptoms for study by first excluding 8 symptoms that were placed in the list to make the instrument less obviously about menopausal symptoms (diarrhea, persistent cough, upset stomach, sore throat, loss of appetite, menstrual problems, fluid retention, urinary tract/bladder infections). We also excluded one symptom, vaginal dryness, which was not expected to vary with contextual stress.

### Statistical analysis

Exploratory factor analyses were carried out with the 14 remaining symptoms to examine how symptoms grouped in the entire sample. Our assumption was that symptoms clustering with hot flashes were our best candidates for the study of midlife symptoms. Scree plots were examined to identify the point at which eigenvalues began to level off. It was decided that three was the most informative number of factors. Three factors were extracted using the method of unweighted least squares with varimax rotation. Unweighted least squares was applied to achieve more conservative results (i.e., fewer symptoms with factor scores > 0.300).

We repeated the factor analyses and each time excluded one symptom that did not cluster with hot flashes. In this way, difficulty concentrating, rapid heartbeat, dizzy spells, and cold sweats were excluded. With each change, the total variance explained increased. The final 10 symptoms were: lack of energy, feeling blue/depressed, backaches, headaches, aches/stiffness in joints, shortness of breath, hot flashes, trouble sleeping, nervous tension, and pins/needles in hands/feet. With fewer than 10 symptoms, the total variance explained started to decline.

Bivariate Spearman correlations were examined among the scores for neighborhood disorder, violence, and discrimination. Spearman correlations were applied because scores were not normally distributed. We examined race/ethnicity in relation to neighborhood characteristics, and each symptom in relation to neighborhood characteristics using Mann-Whitney U tests. Symptoms significantly associated with neighborhood characteristics at the level of *p* < 0.20 were chosen for logistic regression analyses.

In those occasional situations where a participant was missing a subset of the items used to compute a scale score, we used a regression-based approach to estimate the expected value of the scale based on the non-missing items, and replaced/imputed the missing value with its expected value if the R^2^ for the regression > 70%. By definition, the resulting equation is the optimal linear function of the available items for estimating the scale score based on data from those who answered all items.

We examined race/ethnicity, smoking, menopausal status, and education (< 12, 13–16, > 16 years) in relation to each symptom by chi-square analysis, and age and BMI in relation to each symptom by t-test and included race/ethnicity, smoking, education, and/or BMI as covariates in logistic regression models if the relationship between the variable and the symptom was *p* < 0.20 in unadjusted analyses. We did not include all possible covariates in our models in order to increase the power of each model.

Logistic regression analyses were carried out with the symptom (yes/no) as the dependent variable, with each neighborhood or discrimination scale as the primary independent variable controlling for any significant covariate(s). In addition, linear regression was used to examine derived factor scores as outcome variables with each neighborhood or discrimination scale as a predictor variable while controlling for covariates. All analyses were conducted with IBM SPSS Statistics for Windows, Version 24.0. Armonk, NY: IBM Corp.

## Results

Table 1 shows the sample characteristics for the total sample (*n* = 139), and for the white (45%) and black (55%) women. Mean age was 49.1 years and did not vary by race/ethnicity. White women had higher levels of education, but did not significantly differ with regard to smoking, BMI, or menopausal status. All of the neighborhood and discrimination scales differed by race/ethnicity so that black women reported higher scores on negative neighborhood characteristics and discrimination, and a lower score on the positive neighborhood social cohesion and trust scale.

**Table 1:** Sample characteristics

	Total sample	White women	Black women	*p*-value
	*N* = 139	*N* = 62	*N* = 77	
Mean age (s.d.)	49.1 (5.7)	49.7 (6.0)	48.7 (5.5)	0.31
% Level of education				
< 12	16.3%	6.7%	24.6%	< 0.001
13–16	58.9%	50.0%	66.7%	
17+	24.8%	43.3%	8.7%	
% Smoking	22.1%	15.0%	28.2%	0.07
Mean BMI (s.d.)	29.6 (6.4)	28.5 (6.1)	30.6 (6.5)	0.06
% Menopause status				
Pre-	48.0%	39.6%	55.6%	0.09
Peri-	6.9%	4.2%	9.3%	
Post-	45.1%	56.3%	35.2%	
Neighborhood Disorder				
Scale range 0–28				
Means (s.d.)	8.3 (7.4)	5.1 (5.6)	11.1 (7.6)	< 0.001
Medians	6.00	3.23	10.00	< 0.001
Exposure to Violence				
Scale range 0–15				
Means (s.d.)	1.9 (3.0)	0.8 (1.5)	2.9 (3.5)	< 0.001
Medians	1.00	0.00	2.00	< 0.001
Perceived Violence				
Subscale range 5–18				
Means (s.d.)	9.4 (3.6)	8.4 (3.1)	10.4 (3.8)	0.002
Medians	9.00	7.10	10.25	0.004
Neighborhood Social Cohesion and Trust				
Scale range 1–14				
Means (s.d.)	8.4 (2.5)	9.3 (1.9)	7.6 (2.7)	< 0.001
Medians	8.63	10.00	8.00	< 0.001
Everyday Discrimination				
Scale range 0–39				
Means (s.d.)	8.7 (8.2)	6.2 (5.9)	11.1 (9.4)	0.001
Medians	6.00	5.00	7.50	0.002

There were no significant differences between white and black women with regard to symptom report. Only nervous tension approached significance (*p* = 0.05) (Table 2).

**Table 2:** Frequency of symptoms by race/ethnicity

	Total sample	White women	Black women	*p*-value
	(*n*), %	(*n*), %	(*n*), %	
Lack of energy	(66), 55.0%	(31), 57.4%	(35), 53.0%	0.632
Feeling blue/ depressed	(42), 34.7%	(18), 32.7%	(24), 36.4%	0.676
Backaches	(58), 47.9%	(23), 42.6%	(35), 52.2%	0.291
Headaches	(66), 53.7%	(27), 49.1%	(39), 57.4%	0.361
Aches/stiffness in joints	(71), 58.7%	**(27), 50.9%**	**(44), 64.7%**	0.127
Shortness of breath	(22), 18.2%	(8), 14.8%	(14), 20.9%	0.389
Hot flashes	(49), 39.8%	**(18), 33.3%**	**(31), 44.9%**	0.192
Trouble sleeping	(58), 47.5%	**(30), 55.6%**	**(28), 41.2%**	0.114
Nervous tension	(42), 34.4%	**(24), 43.6%**	**(18), 26.9%**	0.052
Pins and needles in hands/feet	(30), 24.4%	**(10), 18.2%**	**(20), 29.4%**	0.149

Differences with a *p* value < 0.20 bolded for inclusion as a covariate in logistic regressions

### Factor analysis

After selecting the 10 symptoms of interest, among all women, the first factor comprised psychosomatic symptoms. Hot flashes loaded onto the second factor along with three somatic symptoms (backaches, aches/stiffness in joints, and pins/needles in hands/feet). A third factor captured some remaining somatic symptoms, including headaches and shortness of breath. Although sample sizes were small, there were differences in factor loadings between white and black women. White women reflected the total sample findings. Among black women, hot flashes clustered with lack of energy, feeling blue/depressed, backaches, and nervous tension in addition to aches/stiffness in joints and pins/needles in hands/feet. Of the 10 symptoms in Table 3, headaches, shortness of breath, and trouble sleeping did not group into a factor with hot flashes (Table 3).

**Table 3:** Factor analyses of symptoms included in study

	Total sample			White women			Black women		
	1	2	3	1	2	3	1	2	3
Lack of energy	.712	.252	.270	.739	.160	.326	.712	.367	−.012
Feeling blue/ depressed	.842	.084	−.144	.776	−.073	−.071	.533	.490	.366
Backaches	.344	.510	.352	.326	.741	−.212	.533	.544	−.182
Headaches	−.058	.040	.803	.104	.098	.609	.162	−.020	−.844
Aches/stiffness in joints	.119	.808	.221	.093	.828	.186	.761	.030	.016
Shortness of breath	.280	.190	.467	−.005	.111	.775	.072	.801	−.051
Hot flashes	.055	.720	−.283	−.152	.631	.221	.485	−.097	.557
Trouble sleeping	.653	.060	.211	.752	.194	−.046	.205	.720	.073
Nervous tension	.717	.250	.002	.597	.045	.456	.713	.283	.329
Pins and needles in hands/feet	.359	.493	.157	.315	.377	.248	.652	.209	−.108
Variance explained (rounded)	25%	18%	13%	23%	19%	15%	29%	20%	13%
Cumulative variance explained	56.31%			56.70%			61.78%		

### Spearman correlations

The neighborhood scales were correlated with each other in the expected directions. Neighborhood Disorder correlated positively with Exposure to Violence (*r* = .649, *p* < 0.001), Perceived Violence (*r* = .679, *p* < 0.001), and Everyday Discrimination Scale (*r* = .495, *p* < 0.001), and correlated negatively with Neighborhood Social Cohesion and Trust (*r* = −.300, *p* = 0.001). Exposure to Violence correlated positively with Perceived Violence (*r* = .489, *p* < 0.001) and Everyday Discrimination Scale (*r* = .422, *p* < 0.001), and negatively with Neighborhood Social Cohesion and Trust (*r* = −.232, *p* = 0.009). Neighborhood Social Cohesion and Trust correlated negatively with Perceived Violence (*r* = −.283, *p* = 0.002) and Everyday Discrimination Scale (*r* = −.318, *p* < 0.001).

### Bivariate results

The following associations were found between symptoms and women’s characteristics (data not shown). With regard to age at interview, women reporting aches/stiffness in joints (*p* < 0.001), hot flashes (*p* < 0.001) and nervous tension (*p* = 0.04) were older than women not reporting those symptoms. Women reporting headaches were younger (*p* = 0.006) than women not reporting headaches. With regard to menopausal status, peri- and post-menopausal women (combined) were more likely to report a lack of energy (*p* = 0.007), aches/stiffness in joints (*p* = 0.004), and hot flashes (*p* < 0.001). Finally, with regard to BMI, women with aches/stiffness in joints (*p* = 0.06), and backaches (*p* = 0.07) tended to have a higher BMI than women not reporting those symptoms. No symptom frequencies differed by smoking status or level of education (< 12, 13–16, > 16 years) at *p* < 0.20.

Looking across bivariate results for symptoms (yes/no) in relation to measures of neighborhood disorder, violence, cohesion, and discrimination, Table 4 shows that the measures of Neighborhood Disorder were associated with 7 symptoms at the *p* < 0.2 level. In all instances, women with the symptoms had higher median levels of neighborhood disorder. The two measures of neighborhood violence (Exposure to Violence and Perceived Violence) were associated with 4 and 3 symptoms, respectively, at the *p* < 0.20 level. The Neighborhood Social Cohesion and Trust was associated with 2 symptoms so that women with more social cohesion and trust in the neighborhood were less likely to report backaches (*p* < 0.05) and aches/stiffness in joints (*p* < 0.20). The Everyday Discrimination Scale was associated with 4 symptoms at the *p* < 0.20 level.

**Table 4:** Median level of each scale by symptom occurrence (yes/no)

	Lack of energy		Feeling blue/ depressed		Backaches		Headaches		Aches/ stiffness in joints		Short of breath		Hot flashes		Trouble sleeping		Nervous tension		Pins and needles in hands/feet	
	No	Yes	No	Yes	No	Yes	No	Yes	No	Yes	No	Yes	No	Yes	No	Yes	No	Yes	No	Yes
ND	*5.09*	*6.00* ^&^	*5.00*	*9.00* ^*^	6.00	6.00	*4.50*	*8.00* ^#^	*4.00*	*8.00* ^#^	*5.04*	*9.00* ^#^	*5.04*	*9.00* ^*^	5.54	6.00	6.00	6.00	*5.09*	*10.00* ^*^
EV	*0.00*	*1.00* ^&^	*0.00*	*1.00* ^&^	1.00	1.00	1.00	1.00	0.00	1.00	1.00	1.00	*0.50*	*2.00* ^&^	1.00	1.00	1.00	1.00	*0.00*	*2.00* ^*^
PV	9.00	9.89	9.00	10.13	9.12	9.59	9.00	10.00	*7.58*	*10.40* ^*^	9.16	9.33	*8.43*	*10.00* ^#^	*10.18*	*9.08* ^&^	9.00	10.13	9.00	10.00
NSCT	8.00	8.51	8.00	9.00	*9.00*	*8.00* ^#^	8.00	8.57	*9.00*	*8.00* ^&^	8.57	8.00	8.30	8.00	8.79	8.00	8.00	8.51	8,00	8.03
EDS	*5.00*	*7.00* ^*^	6.00	7.00	6.00	7.00	*5.00*	*7.00* ^&^	*6.00*	*7.00* ^*^	6.50	6.50	7.00	6.50	6.00	7.50	6.00	8.00	*6.00*	*9.00* ^#^

*p < 0.05; ^#^*p* < 0.1; ^&^*p* < 0.20 using Mann-Whitney U test

*ND* Neighborhood Disorder Scale, *EV* Exposure to Violence Scale, *PV* Perceived Violence Scale, *NSCT* Neighborhood Social Cohesion and Trust, *EDS* Everyday Discrimination Scale

### Logistic regression results

Neighborhood Disorder remained significantly associated with feeling blue/depressed, aches/stiffness in joints, and hot flashes (OR 1.084, 95% CI 1.007–1.165) after controlling for model-specific independent variables (Table 5). Exposure to Violence did not remain associated with any symptom (Table 6), but aches/stiffness in joints remained associated with Perceived Violence after controlling for age, race/ethnicity, BMI and menopausal status (Table 7). There was a lower risk of backaches as the neighborhood cohesion score increased (Table 8). Finally, discrimination (Everyday Discrimination Scale) remained associated with lack of energy after controlling for model-specific independent variables (Table 9).

**Table 5:** Logistic regression results for Neighborhood Disorder (ND)^a^

	Lack of energy	Feeling blue/ depressed	Headaches	Aches/ stiffness in joints	Short of breath	Hot flashes	Pins and needles in hands/feet
	AOR^f^ (95% CI)	AOR (95% CI)	AOR (95% CI)	AOR (95% CI)	AOR (95% CI)	AOR (95% CI)	AOR (95% CI)
Age	.92 (.82–1.03)		*.86 (.77–.96)* ^c^	1.13 (.99–1.28)		1.05 (.94–1.18)	1.04 (.97–1.13)
Black				2.62 (.89–7.73)		1.70 (.62–4.69)	1.41 (.54–3.68)
BMI	1.04 (.97–1.12)			1.04 (.955–1.126)	1.04 (.06–1.12)		
Peri/Post^b^	*7.32 (1.80–29.76)* ^d^		1.88 (.55–6.38)	2.66 (.62–11.36)		*4.73 (1.25–17.93)* ^e^	
ND	1.07 (.997–1.145)	*1.07 (1.02–1.13)* ^f^	1.06 (.99–1.12)	*1.11 (1.01–1.21)* ^g^	1.06 (.997–1.13)	*1.08 (1.01–1.17)* ^h^	1.06 (.99–1.13)

^a^Symptoms selected for logistic regression were those associated with the neighborhood characteristic in Table 4

^b^Pre is the reference

^c^*p* = .007; ^d^*p* = .005; ^e^*p* = .022; ^f^*p* = 0.011; ^g^*p* = 0.030; ^h^*p* = 0.031

^f^*AOR* adjusted odds ratio

**Table 6:** Logistic regression results for Exposure to Violence (EV)^a^

	Lack of Energy	Feeling blue/depressed	Hot flashes	Pins and needles in hands/feet
	AOR^c^ (95% CI)	AOR (95% CI)	AOR (95% CI)	AOR (95% CI)
Age	.93 (.83–1.04)		1.07 (.96–1.20)	1.05 (.97–1.13)
Black			2.02 (.74–5.47)	1.47 (.57–3.77)
BMI	1.05 (.97–1.13)			
Peri/Post^a^	*5.82 (1.52–22.38)* ^d^		*3.61 (1.01–12.92)* ^e^	
EV	1.13 (.96–1.33)	1.06 (.94–1.20)	1.11 (.95–1.304)	1.13 (.99–1.30)

^a^Symptoms selected for logistic regression were those associated with the neighborhood characteristic in Table 4

^b^Pre is the reference

^c^*AOR* adjusted odds ratio

^d^*p* = 0.010; ^e^*p* = 0.048

**Table 7:** Logistic regression results for Perceived Violence (PV)^a^

	Aches/ stiffness in joints	Hot flashes	Trouble sleeping
	AOR^e^ (95% CI)	AOR (95% CI)	AOR (95% CI)
Age	1.13 (.99–1.29)	1.05 (.93–1.18)	
Black	2.55 (.85–7.63)	2.54 (.90–7.20)	.69 (.31–1.54)
BMI	.998 (.91–1.09)		
Peri/Post^b^	1.57 (.35–7.05)	*4.27 (1.09–16.68)* ^c^	
PV	*1.17 (1.01–1.36)* ^d^	1.10 (.96–1.25)	.95 (.85–1.06)

^a^Symptoms selected for logistic regression were those associated with the neighborhood characteristic in Table 4

^b^Pre is the reference

^c^*p* = 0.037; ^d^*p* = 0.035

^e^*AOR* adjusted odds ratio

**Table 8:** Logistic regression results for the Neighborhood Social Cohesion and Trust (NSCT) Scale^a^

	Backaches	Aches/ stiffness in joints
	AOR^*^ (95% CI)	AOR (95% CI)
Age	.	*1.14 (1.01–1.29)* ^c^
Black		*3.40 (1.16–9.97)* ^d^
BMI	1.05 (.98–1.12)	1.03 (.95–1.12)
Peri/Post^b^		2.03 (.49–8.46)
NSCT	*.85 (.72–.99)* ^e^	.94 (.78–1.13)

^*^AOR adjusted odds ratio

^a^Symptoms selected for logistic regression were those associated with the neighborhood characteristic in Table 4

^b^Pre is the reference

^c^*p* = 0.042; ^d^*p* = 0.026; ^e^*p* = 0.035

**Table 9:** Logistic regression results for Everyday Discrimination Scale (EDS)^a^

	Lack of energy	Headaches	Aches/ stiffness in joints	Pins and needles in hands/feet
	AOR^*^ (95% CI)	AOR (95% CI)	AOR (95% CI)	AOR (95% CI)
Age	.92 (.82–1.04)	*.87 (.78–.97)* ^c^	1.14 (.998–1.29)	1.05 (.97–1.13)
Black			2.75 (.94–8.07)	1.80 (.71–4.55)
BMI			1.03 (.95–1.12)	
Peri/Post^b^	*8.07 (1.93–33.81)* ^d^	1.63 (.48–5.54)	2.26 (.51–9.98)	
EDS	*1.10 (1.03–1.18)* ^e^	1.05 (.99–1.11)	1.08 (.996–1.17)	1.05 (.997–1.11)

^*^AOR adjusted odds ratio

^a^Symptoms selected for logistic regression were those associated with the neighborhood characteristic in Table 4

^b^Pre is the reference

^c^*p* = 0.010; ^d^*p* = 0.004; ^e^*p* = 0.006

Looking across Tables 5, 6, 7, 8 and 9, in addition to neighborhood context and discrimination, increasing age reduced the risk of headaches, but elevated the risks of aches/stiffness in joints. Peri/post-menopausal status was associated with an increased likelihood of lack of energy in two models (OR 7.324 and OR 8.071) and an increased likelihood of hot flashes in three models (OR 4.734, OR 3.611, and OR 4.265). BMI was not associated with any symptom in logistic regression models after controlling for age, race/ethnicity, menopausal status, and neighborhood characteristics.

### Linear regression results

Both Neighborhood Disorder and Everyday Discrimination scores were significantly associated with derived Factor 1 scores (data not shown). Symptoms loading onto Factor 1 included “Feeling blue or depressed” and “Lack of energy.” These results are consistent with our logistic regression results where the associations were significant between “Feeling blue or depressed” and Neighborhood Disorder (*p* = 0.011) and between “Lack of energy” and Everyday Discrimination (*p* = 0.006).

## Discussion

The results of this study suggest that neighborhood context and discrimination may be associated with midlife symptoms in a cohort of black and white women residing in a large urban environment. To our knowledge, this is one of very few studies to extend the investigation of perceived social features of neighborhoods to symptoms among women at midlife. A major strength of this study is the many measures of neighborhood context collected in relation to the broad range of symptoms examined. A novel approach used factor analysis to focus our examination on ten symptoms, clustered on three factors. Of those ten symptoms, five were found to be significantly associated with neighborhood context or discrimination.

As is often the case with midlife symptoms [58–61], the ten symptoms of interest did not separate cleanly into distinct groups through factor analyses. Also consistent with other studies [58, 61], there is population variation in how symptoms cluster. In the study presented here, depressed mood and hot flashes grouped together among the Black sample, but not the White sample or in the sample as a whole.

Lack of energy, feeling blue/depressed, aches/stiffness in joints, and hot flashes were the symptoms most vulnerable to the effect of negative neighborhood context. Each of these four symptoms remained significantly associated with different neighborhood characteristics after adjusting for model-specific covariates. All but lack of energy were associated with Neighborhood Disorder.

Why these symptoms would be most affected by neighborhood context is not immediately clear. Looking at the factor analyses, aches/stiffness in joints consistently clustered together with hot flashes, but feeling blue/depressed only clustered with hot flashes among Black women. Backaches and pins/needles also clustered with hot flashes, but backaches were only significantly associated with the Neighborhood Social Cohesion and Trust, and pins/needles were not associated with any measure of neighborhood context or discrimination. The factor analyses did not help us predict how symptoms would be associated with neighborhood stress.

Neighborhood Disorder remained significantly associated with feeling blue/depressed, aches/stiffness in joints, and hot flashes after controlling for model-specific independent variables. This suggests that stress related to neighborhood disorder may be expressed as emotional, somatic, and vasomotor experience. Somatization of emotional symptoms may at times serve as psychosomatic “idioms of distress,” calling attention to difficulties that are hard to verbally express [62–64].

Aches/stiffness in joints remained associated with Perceived Violence, but not Exposure to Violence, after controlling for age, race/ethnicity, BMI and menopausal status. Because of differences in the scales, as well as the relatively modest correlation between them (*r* = .489), it is not surprising that they are not similarly associated with midlife symptoms.

Only backaches were associated with the neighborhood cohesion score, decreasing the risk of backaches as the score increased (Table 8). Women were 15% less likely to report having had backaches for each unit increase (1 point on a 0–15 point scale) on the Neighborhood Social Cohesion and Trust scale. Backaches may also be indicative of depression and somatization [65, 66]. The Multi-Ethnic Study of Atherosclerosis (MESA) found that neighborhoods with low levels of social cohesion had increased rates of depression [37], but that was not the case here. It should be noted that the Neighborhood Social Cohesion and Trust scale in the MESA study was evaluated as tertiles, and a different measure of depressed mood (the Centers of Epidemiologic Studies Depression scale) was used.

Finally, the Everyday Discrimination Scale remained associated with lack of energy after controlling for model-specific independent variables (Table 9). A number of studies have documented associations between discrimination and physical symptoms The SWAN study found everyday discrimination was significantly associated with bodily pain in all ethnic groups [47]. In contrast, data from the Midlife Development in the United States study (MIDUS) did not show a significant association in whites, but demonstrated a significant positive relationship between perceived discrimination and frequency of back pain among African Americans, with a stronger association observed among African-American women [67].

In examining the relation between neighborhood social environments and discrimination with midlife symptoms, there were also contributions of age, race/ethnicity, and menopausal status. Older women had a reduced risk of headaches compared to younger women, in contrast to studies of tension-related headaches [68]. Older women had an elevated risk of aches/stiffness in joints, as would be expected [69]. Although higher rates of reported pain among African-Americans was noted in Dugan et al. [47], the differences of 65% among black women vs 50% among white women in the frequency of aches/stiffness of joints observed in this study did not reach statistical significance, perhaps due to small numbers. Nervous tension was reported less frequently among black than white women (27% vs 44%, respectively), approaching statistical significance (*p* = 0.052). It is interesting to note, however, in models where neighborhood context or discrimination were significantly associated with a midlife symptom, race/ethnicity did not significantly add risk.

Peri/post-menopausal status was associated with an increased likelihood of lack of energy and, in three models, an increased likelihood of hot flashes. Lack of energy is frequently one of the most commonly reported symptoms among women at menopause [56, 70, 71], and it is well established that the loss of estrogen during the late menopausal transition is associated with hot flashes [1].

This study has several limitations. There is limited assessment of symptoms (i.e., presence in past 2 weeks, without assessment of frequency or bothersomeness.) Given the cross-sectional design of this study, we are unable to determine the temporal sequence of the reported relationships. This study posited that the effects of neighborhood context would be related to symptoms at midlife; however, a depressed person might perceive her neighborhood more negatively than a person without depressive symptoms [72]. Longitudinal studies are needed to address the direction of any causal association.

There is a potential for spurious associations given the multiplicity of outcomes. We were careful, however, to limit just those covariates into multivariable models that were significant or marginally significant in bivariate analyses. An additional limitation is the potential lack of power to detect significant findings due to the small sample size. Among the strengths of this study is the fact that participants were drawn from four distinct sites from a large and diverse city. Additionally, standardized data collection protocols were used across sites to assess multiple measures of neighborhood characteristics.

## Conclusions

This study adds to the literature linking neighborhood environments to health outcomes. We have extended this literature to a number of women’s midlife symptoms that have not been previously examined. In our sample comprised of both black and white women, we found that negative neighborhood context increased the risk of self-reported depression, aches/stiffness in joints, and hot flashes while greater social cohesion lowered the risk of backaches. The association between discrimination and lack of energy is intriguing, and points to the need to further examine links between exposure to everyday discrimination, and other measures of neighborhood context, and multiple physiological systems.
